# Risk Factors for COVID-19 in Patients with Hypertension

**DOI:** 10.1155/2021/5515941

**Published:** 2021-05-07

**Authors:** Xinxin Wang, Haihua Zhang, Huan Du, Ruina Ma, Yandong Nan, Tao Zhang

**Affiliations:** ^1^Department of Pulmonary and Critical Care Medicine, Tangdu Hospital, Air Force Medical University, Xi'an, China; ^2^Wuhan Huoshenshan Hospital, Wuhan, China

## Abstract

**Background:**

Hypertension, as the most common comorbidity for patients with coronavirus disease 19 (COVID-19), has resulted in cases with more severe symptoms and higher mortality. The risk factors associated with COVID-19 in patients with hypertension are unknown.

**Methods:**

All the available and confirmed patients with COVID-19 from February 3 to March 10, 2020, were enrolled from Huoshenshan Hospital, Wuhan, China. The demographic characteristics, clinical manifestations, laboratory data, radiological assessments, and treatments on admission were extracted and compared. Univariate and multivariate logistic regression methods were used to explore risk factors associated with COVID-19 in patients with hypertension and the severity of the cohort.

**Results:**

A total of 430 available patients with COVID-19 were enrolled in the study, including 151 eligible patients with COVID-19 and hypertension. After PSM analysis, 141 patients without hypertension and 141 cases with hypertension were well matched. Compared with cases without hypertension, patients with hypertension were more severe (28.4% vs. 12.1%, *p*=0.001). In multivariate analysis, we found that neutrophil count (OR: 1.471; *p*=0.001), coronary heart disease (OR: 5.281; *p*=0.011), and the level of K^+^ (OR: 0.273; *p* < 0.001) were associated with patients with hypertension. In addition, the percentage of pulmonary infection volume was larger in cases with hypertension (4.55 vs. 5.8, *p*=0.017) and was a high risk factor for severe COVID-19 in patients with hypertension (OR: 1.084; *p* < 0.001).

**Conclusion:**

On admission, coronary heart disease, neutrophil count, and the level of K^+^ were associated with COVID-19 patients with hypertension. The percentage of the pulmonary infection volume was significantly larger in COVID-19 patients with hypertension and was a risk factor for COVID-19 severity of the cohort.

## 1. Introduction

The recently emerged severe acute respiratory syndrome coronavirus 2 (SARS-CoV-2), causing coronavirus disease 19 (COVID-19), poses a serious global health emergency because of its high pathogenicity and rapid international spread. Hypertension is the most common comorbidity for patients with COVID-19. A large number of studies have highlighted the overrepresentation of hypertension and the much higher fatality among patients with hypertension hospitalized with COVID-19 [1, 2]. Among 20982 patients confirmed to have COVID-19, 12.6% suffered from hypertension, which was the most frequent coexisting condition, and the overall proportion of hypertension was 39.75% in 406 deceased COVID-19 patients [3]. In another epidemiological study of an outbreak of COVID-19 from China, the estimated prevalence of patients with hypertension was up to 12.8% among 44672 patients confirmed to have COVID-19, and the patients were more likely to develop serious symptoms, with a mortality rate of 6.0% [4]. In a report concerning 12226 patients who required hospital admission in 150 Spanish centers, hypertension (50.9%) was the most common comorbidity and the preexisting condition of hypertension had an independent prognostic value for all-cause mortality in patients with COVID-19 [5].

SARS-CoV-2 is a new member of the beta-coronavirus genus and can recognize ACE2 on the lung alveolar epithelial cells as its host entry receptor, resulting in acute pulmonary infection [6]. Renin-angiotensin-aldosterone system (RAAS) inhibitors, including angiotensin-converting enzyme inhibitors (ACEIs) and angiotensin receptor blockers (ARBs), as one of the most important antihypertensive therapies, might upregulate the expression of ACE2 and thus facilitate the deterioration of patients' condition during the ongoing COVID-19 pandemic [7]. A large number of clinical studies and meta-analyses focused on antihypertensive therapies and have showed that RAAS inhibitors were beneficial to treat acute lung injury during severe acute respiratory syndrome coronavirus 2 (SARS-CoV-2) infection. The risk factors associated with COVID-19 patients with hypertension were still unknown.

In addition, chest radiological manifestation can reflect the actual lung infection objectively. As yet, no study uses quantifiable parameters on CT findings to assess the lung infection extent of COVID-19 patients with hypertension.

In the present study, we described the clinical characteristics of 282 COVID-19 patients with and without hypertension using the propensity score-matching (PSM) method to investigate the risk factors for COVID-19 in patients with hypertension. Besides, through comparing severe cases with nonsevere cases, 151 patients with COVID-19 and hypertension were included to explore risk factors associated with the severity of COVID-19 in patients with hypertension. The lung infection percentage, which was calculated automatically using a deep learning-based software, was included. Our findings will provide guidance for the early administration of patients with COVID-19 and hypertension to prevent their further deterioration.

## 2. Methods

### 2.1. Participants

This single-center, retrospective study was conducted in Huoshenshan Hospital of Wuhan, in China, one of the makeshift hospitals dedicated to treating patients infected with the deadly coronavirus, which was constructed in one week, with a capacity of 1,000 beds, and formally delivered to military medics on February 2, 2020.

All patients with confirmed COVID-19 and hypertension, diagnosed according to the guidance provided by the fifth version of the guidelines issued by the National Health Commission of China on Diagnosis and Treatment of COVID-19 and the 2018 guidelines of the European Society of Hypertension (ESH) [8, 9] (Supplementary Table 1), were enrolled in our study, from February 3 to March 10, 2020. Pediatric patients and cases with an unclear history of the illness were excluded from the study. For discharged patients, we directly communicated with the patients or their families to ascertain their living status up to March 10, 2020.

The Ethics Commission of Huoshenshan Hospital approved this study. Informed consent was waived for this retrospective study.

### 2.2. Data Collection

The demographic characteristics, clinical symptoms, laboratory data, radiological assessments, and medications were extracted from electronic medical records and via telephone conversation for some unclear conditions, by a trained team of physicians. Two researchers then independently reviewed the collected data in case record forms and a third researcher judged any disagreements.

All the laboratory and radiological examinations were performed at Huoshenshan Hospital on admission, except for the nucleic acid test for SARS-CoV-2. From the 3rd to the 20th of February, respiratory specimens were sent to the local Centers for Disease Control and Prevention for SARS-CoV-2 nucleic acid detection using next-generation sequencing or real-time reverse transcription PCR methods. After February 20, testing for SARS-CoV-2 nucleic acid was performed at Huoshenshan Hospital using real-time RT-PCR methods.

**Figure 1:**
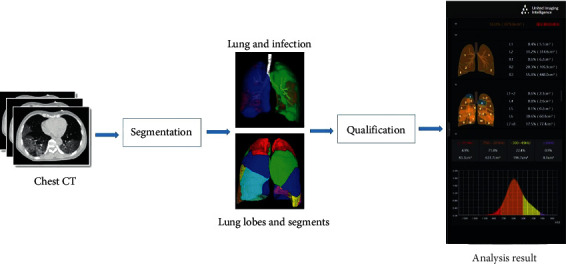
A flowchart for calculating the percentages of infection in the whole lung [10].

Chest computed tomography (CT) was performed for all eligible inpatients. For the radiological examinations, not only were traditional descriptions including lesion shape, texture, and size reported, but also the infection volume ratio, which was calculated automatically using artificial intelligence (AI) software (United Imaging Intelligence, Shanghai, China), which could assess the severity of lung infection more precisely. There is a segmentation system in the software. After the baseline CT scans of patients were fed to the system, various metrics were computed to quantify the COVID-19 infection, including the percentages of infection in the whole lung (Figure 1) [10].

### 2.3. Definitions

Patients were divided into a severe group and a nonsevere group according to the fifth version of the guidelines issued by the National Health Commission of China on Diagnosis and Treatment of COVID-19 [8].

COVID-19 severity is classified as follows:

1. Mild type: the clinical symptoms are mild with no abnormal radiological findings
2. Moderate type: fever, cough, and other symptoms are presented with pneumonia on chest computed tomography
3. Severe type: one of the following conditions is met: (1) respiratory distress and respiratory rate ≥30 per min; (2) oxygen saturation on room air at rest ≤93%; and (3) partial pressure of oxygen in arterial blood/fraction of inspired oxygen ≤300 mmHg
4. Critical type: one of the following conditions is met: (1) respiratory failure occurs and mechanical ventilation is required; (2) shock; and (3) patients with other organ dysfunction needing intensive care unit monitoring treatment

For limited critical cases with incomplete clinical data, severe, moderate, and mild types of COVID-19 patients were finally included in the study.

Hypertension was classified as Grade 1, Grade 2, and Grade 3 according to the 2018 guidelines of the European Society of Hypertension (ESH) [9] (Supplementary Table 1). Fever was considered as an axillary temperature above 37.2°C. Shock was defined according to the Third International Consensus Definitions for Sepsis and Septic Shock (Sepsis-3) in 2016 [11]. The date of disease onset was defined as the day when the related symptoms appeared or abnormal chest radiological findings were observed.

### 2.4. Statistical Analysis

In the statistical analysis, categorical variables expressed as proportions were compared using the chi-squared test and Fisher's exact test. Continuous variables, shown as the median with the interquartile range (IQR) values for nonnormally distributed data or as the means ± standard deviation (SD) for normally distributed data, were compared using an independent group *t*-test or the nonparametric Mann–Whitney *U* test, when appropriate.

PSM analysis was performed to minimize the effect of selection bias and to control for potential confounding factors. It is well known that older people have a higher likelihood to have hypertension. Age and sex matching between COVID-19 cohort with and without hypertension were conducted using a 1 : 1 matching protocol with caliper width = 0.2 of the standard deviation of the propensity score.

Univariate and multivariate logistic regression methods were used to explore risk factors associated with the severity of COVID-19 in patients with hypertension. In the multivariate logistic regression, variables were excluded if their between-group differences were not significant from the univariate analysis. However, in the multivariate logistic regression of risk factors for severe cases with hypertension, considering the total number of patients with severe disease (*n* = 45), and to avoid overfitting, age, CK-MB (creatine kinase isoenzyme), ACEIs/ARBs treatment, platelet count, percentage of the infected region, and calcium ions were chosen for multivariate regression modeling using a backward stepwise model. The following factors were considered. Calcium channel blockers (CCB), as antihypertensive drugs, might influence the level of platelets and calcium ions. Coronary heart disease was the common complication for patients with hypertension, so CK-MB, as one of the cardiac biomarkers after troponin I, which was not significant from the univariate analysis, was included. In addition, previous studies had shown older age was a risk factor for poor outcome [12]. *p* < 0.05 was considered statistically significant. All analyses were conducted using IBM SPSS Statistics v 21.0 (IBM Corp., Armonk, NY, USA).

## 3. Results

**Figure 2:**
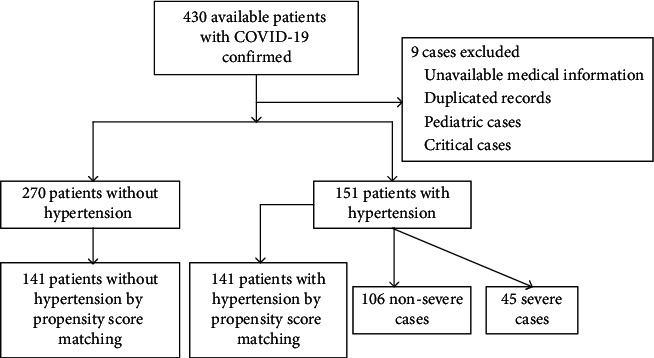
Flow diagram of patient screening and recruitment.

The final analysis included 151 eligible patients with hypertension and COVID-19, including 45 severe and 106 nonsevere cases, from 430 available patients confirmed to have COVID-19 admitted to Huoshenshan Hospital by March 10, 2020. Among them, 9 patients were excluded because they were suspected or pediatric cases, had missing medical information, duplicated records, or critical cases with limited medical records, especially CT scan. After one-to-one PSM analysis, 141 cases from 270 COVID-19 patients without hypertension and 141 COVID-19 patients with hypertension were respectively matched (Figure 2).

Compared with cases without hypertension, patients with hypertension were more severe (28.4% vs. 12.1, *p*=0.001) and were more likely to have a comorbidity of diabetes (19.1% vs. 9.9%, *p*=0.028), coronary heart disease (17.0% vs. 6.4%, *p*=0.005), and cerebrovascular disease (8.5 vs. 2.1, *p*=0.017). Only 20.2% had exposure to the Huanan seafood market. The most common symptom was fever (76.2%), followed by cough (70.2%) and fatigue (59.9%). Sputum (9.9% vs. 5.0%, *p* < 0.001), headache (39.7% vs. 4.3%, *p* < 0.001), and myalgia (37.6% vs. 19.1%, *p*=0.001) were the most common symptoms at onset of illness for patients without hypertension (Table 1).

In terms of laboratory findings, compared with patients without hypertension, patients with hypertension showed a higher median white blood cell count (6.2 vs. 5.4, *p* < 0.001), especially the neutrophil count (3.3 vs. 4.4), but a lower level of K^+^ (4.2 vs. 4.4, *p*=0.002). There were significant differences in the levels of ALP (68.1 vs. 74.6, *p*=0.019), creatinine (64.6 vs. 68.1, *p*=0.013), uric acid (255.5 vs. 286, *p*=0.034), and urea nitrogen (4.64 vs. 5.12, *p*=0.003) between the two groups of patients. On admission, 271 (96.1%) of the patients showed bilateral involvement on chest CT images. The most common abnormalities for patients with hypertension were multiple mottling opacity (90.1 vs. 55.3, *p* < 0.001) and typical ground-glass opacity (84.4 vs. 63.8, *p* < 0.001). On admission, patients with hypertension showed a larger percentage of the pulmonary infection volume than patients without hypertension (4.55 vs. 5.8, *p*=0.017) (Table 2 and Supplementary Table 2).

Among 151 patients with COVID-19 and hypertension, a total of 149 cases have primary hypertension, and two patients with nonsevere COVID-19 had secondary hypertension. The median length of hypertension was 10 years. However, 88 patients had suffered from hypertension for too long to recall the duration accurately. In terms of grade of hypertension, 11, 34, and 20 patients were classified as grade one, grade two, and grade three, respectively. Calcium channel blockers (CCBs) had been administered to 85 patients. Patients with nonsevere disease were more likely to require ACEIs/ARBs (24.5% vs. 8.9%, *p*=0.031), especially for ARBs (18.9% vs. 2.2%, *p*=0.007), which was the second most popular antihypertensive treatment (Table 3).

Before the multivariate logistic analysis, univariate analysis was performed to screen out the appropriate variables. After univariate analysis, diabetes, coronary heart disease, cerebrovascular disease, decreased platelet count and calcium ions, elevated white blood cell count, neutrophil count, ALP, urea nitrogen, uric acid, creatinine, and decreased K^+^ had effects on COVID-19 in patients with hypertension. In multivariate analysis, We found that neutrophil count (OR: 1.471, *p*=0.001), coronary heart disease (OR: 5.281, *p*=0.011), and the level of K^+^ (OR: 0.273, *p* < 0.001) were high risk factors for COVID-19 in patients with hypertension (Tables 4 and 5).

In univariate analysis for 151 patients with hypertension, age, ACEIs/ARBs, lymphopenia, leucocytosis, percentage of the pulmonary infection volume, decreased platelet count and calcium ions, and elevated white blood cell count, neutrophil count, lactate dehydrogenase (LDH), myoglobin, D-dimer, procalcitonin (PCT), and C-reactive protein, AST, albumin, and CK-MB were explored for their association with severe COVID-19 in patients with hypertension (Supplementary Table 3). We found that the percentage of pulmonary infection volume was a high risk factor for severe COVID-19 in patients with hypertension (OR: 1.084, *p* < 0.001) (Table 6).

## 4. Discussion

Hypertension is the most common coexisting medical condition for patients with COVID-19 and is associated with higher mortality. Hypertension usually can mediate target organ damage or cardiovascular disease, which may be determinant factors for COVID-19. Coronary heart disease is the main complication of hypertension. It was reported that cardiac injury was an independent predictive factor for the death of patients with COVID-19 [13]. Patients with preexisting cardiovascular diseases were more susceptible to COVID-19-induced heart injury [14]. In the present study, we also found that coronary heart disease was associated with COVID-19 patients with hypertension. However, there was no difference between patients with hypertension and those without hypertension for high-sensitivity troponin I, which is a specific cardiac indicator, as well as myoglobin and creatine kinase isoenzymes, as cardiac biomarkers. ACE2, as a monocarboxypeptidase glycoprotein and the entry receptor for SARS-CoV-2, was also expressed in the kidney [15]. In one study with 193 COVID-19 patients at hospital admission, more patients had higher rates of AKI, and COVID-19 patients with AKI had a significantly higher mortality risk [16]. In our study, creatinine and urea nitrogen were found to be higher in patients with hypertension, but in univariate analysis, was not associated with COVID-19 cases with hypertension. Our results demonstrated the percentage of pulmonary infection volume was different between COVID-19 patients with hypertension and cases without hypertension, and multivariate regression showed that the percentage of pulmonary infection volume was associated with the severity of COVID-19 in patients with hypertension.

Chest radiological manifestation can reflect the actual lung infection objectively. Usually, a computed tomography scoring system including radiographic features and distributions in two dimensions, which are finished manually, is used to assess the extent of pulmonary pathology in COVID-19 patients [17, 18]. While the scoring system can reflect the CT findings, a quantifiable parameter was still needed. To the best of our knowledge, this is the first report to analyze the percentage of pulmonary infection volume of patients with COVID-19 and hypertension in the hospital. In our study, the lung infection percentage was introduced to assess lung injury in three dimensions, which was calculated automatically using a deep learning-based software, instead of the rough estimate of the infection area obtained from traditional CT. SARS-CoV-2 was more likely to damage the lung tissue of patients with hypertension, leading to increased numbers of severe cases.

Recent understanding of the dysregulation immune system in hypertension may provide a possible explanation why hypertension is potentially associated with a more severe course of COVID-19. SARS-CoV-2 induces the overproduction of proinflammatory cytokines, including interleukin-6 (IL-6), IL-2, IL-7, and tumour necrosis factor-*α* (TNF-*α*), resulting in a “cytokine storm” of overactivated neutrophils, monocyte, and lymphocytes [2]. In experimental and clinical observations, patients with hypertension showed inflammatory dysregulation. Clinical hypertension is undoubtedly associated with immune activation. Increased numbers of central memory CD8+ T cells, which can produce TNF, were found in patients with hypertension [19]. Compared with healthy cases, monocytes from patients with essential hypertension are preactivated and would produce more IL-6 after stimulation with angiotensin II or lipopolysaccharide [20]. The immune dysregulation in patients with hypertension makes the cases to be more prone to be infected with SARS-CoV-2. In addition, in humans, ACE2 is expressed widely in many organs, including the heart, kidney, livers, intestines, and the lung alveolar epithelial cells [19]. However, it was found that 83% of ACE2-expressing cells are type II alveolar cells [21] and the large surface area of the lung makes SARS-CoV-2 particularly vulnerable to inhaled viruses. Therefore, it is reasonable that SARS-CoV-2 was more likely to damage the lung tissue of patients with hypertension, leading to increased numbers of severe cases.

In addition, it was found that neutrophil count patients and the level of K^+^ were risk factors for COVID-19 in patients with hypertension. Neutrophils are a well-known marker of systemic inflammation and infection, usually as a predictor of bacterial infection. COVID-19-induced NKG2A expression may be correlated with functional exhaustion of lymphocytes (including CTLs and NK cells), which may result in severe pulmonary inflammation [22]. Because of the serious disturbance in the immune system, which may also have an effect on the level of K^+^, cases with hypertension would get more susceptible to bacterial infection with neutrophils increased reactively and developed a potentially severe condition.

## 5. Study Limitations

There are some limitations to our study. First, given the retrospective design at a single center, not all laboratory tests were done in all patients and some records of the patients' medical history were missing. Second, there were about 33 from 282 patients with COVID-19 and 28 from 151 COVID-19 cases with hypertension were so severe that they were not able to complete a chest CT. The majority of the above patients had been taken chest radiographs bedside. In multivariable analysis, only cases with chest CT were included. Therefore, the value of the infection volume percentage might be underestimated in severe cases. Third, the sample size of the present study was small; larger populations and multiple centers are warranted to further confirm the outcomes of COVID-19 infection in patients with hypertension.

## 6. Conclusions

Coronary heart disease, neutrophil count patients, and the level of K^+^ were associated with COVID-19 patients with hypertension. The percentage of the pulmonary infection volume was significantly larger in COVID-19 patients with hypertension and was a risk factor for COVID-19 severity of patients with hypertension. Early treatments should focus on lung protection for these cases and prevent bacterial infections if necessary.

## Figures and Tables

**Table 1:** Demographic and clinical characteristics of 282 patients with COVID-19.

Clinical characteristics	All (*n* = 282)	Nonhypertension (*n* = 141)	Hypertension (*n* = 141)	*p* value
Age, median (IQR), years	67 (62–71)	67 (62–71)	5.8 (1.3–12.3)	0.491

Sex				
Female	132 (46.8)	64 (45.4)	68 (48.2)	0.633
Male	150 (53.2)	77 (54.6)	73 (51.8)	

Severe	57 (20.2)	17 (12.1)	40 (28.4)	0.001
Nonsevere	225 (79.8)	124 (87.9)	101 (71.6)	
Exposure history	57 (20.2)	24 (17.0)	33 (23.4)	0.011
Nonexposure history	119 (42.2)	28 (19.9)	91 (64.5)	
Smoking	24 (8.5)	12 (8.5)	12 (8.5)	1

Comorbiditie*s*				
Diabetes	41 (14.5)	14 (9.9)	27 (19.1)	0.028
Carcinoma	5 (1.8)	0	5 (3.5)	0.075
Coronary heart disease	33 (11.7)	9 (6.4)	24 (17.0)	0.005
Cerebrovascular disease	15 (5.3)	3 (2.1)	12 (8.5)	0.017
Chronic pulmonary disease	5 (1.8)	5 (3.5)	0	0.071
Kidney disease	10 (3.5)	4 (2.8)	6 (4.3)	0.52
Others	51 (18.1)	26 (18.4)	25 (17.7)	0.877

Symptoms				
Fever	215 (76.2)	113 (80.1)	102 (72.3)	0.124
Cough	198 70.2)	106 (75.2)	92 (65.2)	0.068
Pharyngodynia	5 (1.8)	3 (2.1)	2 (5.0)	1
Sputum	21 (7.4)	14 (9.9)	7 (5.0)	<0.001
Wheezing	92 (32.6)	51 (36.2)	41 (29.1)	0.204
Shortness of breath	58 (20.6)	26 (18.4)	32 (22.7)	0.377
Nausea or vomiting	6 (2.1)	5 (3.5)	10.7 ()	0.216
Abdominal pain	2 (0.7)	1 (0.7)	1 (0.7)	1
Diarrhea	16 (5.7)	9 (6.4)	7 (5.0)	0.607
Chest distress	51 (18.1)	27 (19.1)	24 (17.0)	0.643
Flusteredness	5 (1.8)	3 (2.1)	2 (1.44)	1
Fatigue	169 (59.9)	88 (62.4)	81 (57.4)	0.395
Headache	62 (30.0)	56 (39.7)	6 (4.3)	<0.001
Myalgia	80 (28.4)	53 (37.6)	27 (19.1)	0.001
Dizziness	22 (7.8)	14 (9.9)	8 (5.7)	0.183
Anorexia	11 (3.9)	7 (5.0)	4 (2.8)	0.356
Rhinobyon	3 (1.1)	1 (0.7)	2 (1.4)	1

Vital signs (IQR)				
Respiratory rate, median (IQR), breaths per min	20 (19–21)	20.00 (19.50–21.00)	20 (19–22)	0.867
Heart rate, median (IQR), beats per min	84 (78–90)	84.00 (78.00–91.50)	82 (78–90)	0.37
Temperature, median (IQR),°C	36.6 (36.4–36.9)	36.60 (36.40–36.90)	36.6 (36.4–36.90	0.941
Systolic blood pressure	130.00 (120.00–139.75)	129.50 (120.00–136.25)	130 (121–144)	0.152
Diastolic blood pressure	78.00 (72.00–85.00)	78.0 (70.75–82.0)	79.0 (72.75–87.25)	0.055
Time from illness onset to admission, (IQR), days	14.00 (10.00–18.00)	14.0 (10.0–17.0)	14.0 (10.0–19.5)	0.447

IQR: interquartile range.

**Table 2:** Laboratory and radiographical findings of 282 patients with COVID-19 on admission.

Clinical characteristics	All (*n* = 282)	Nonhypertension (*n* = 141)	Hypertension (*n* = 141)	*p* value
*Laboratory findings, median (IQR)*				
White blood cell count, ×10^9^/L	5.80 (4.50–7.00)	5.40 (4.10–6.80)	6.2 (5.2–7.8)	<0.001
Neutrophil count, ×10^9^/L	3.79 (2.80–5.28)	3.28 (2.37–4.95)	4.42 (3.11–5.89)	<0.001
Monocyte count, ×10^9^/L	0.44 (0.33–0.57)	0.42 (0.32–0.57)	0.45 (0.35–0.57)	0.746
Lymphocyte count, ×10^9^/L	1.18 (0.84–1.65)	1.23 (0.91–1.65)	1.15 (0.77–1.64)	0.219
Haemoglobin, g/L (mean ± SD)	124.00 (113.00–133.00)	123.50 (112.00–131.00)	124 (114–135)	0.214
Platelet count, ×10^9^/L	254.65 (160.31–348.99)	262.47 (173.25–351.69)	246.73 (147.77–345.69)	0.165
Eosinophil count, ×10^9^/L	0.07 (0.03–0.12)	0.06 (0.03–0.11)	0.07 (0.01–0.12)	0.856
Basophils count, 10^9^/L	0.01 (0.01–0.02)	0.01 (0.01–0.02)	0.01 (0.01–0.02)	0.772
C-reactive protein, mg/L	6.03 (1.99–36.53)	5.19 (1.72–18.85)	8.2 (2.62–46.32)	0.067
IL-6, pg/ml	4.91 (2.38–15.62)	5.69 (2.62–96.88)	4.82 (2.38–1.64)	0.534
PCT, ng/ml	0.05 (0.03–0.20)	0.05 (0.03–0.07)	0.06 (0.03–0.41)	0.438
ALT, IU/L^a^	25.35 (17.18–42.45)	25.2 (17.75–39.25)	25.6 (16.75–48.25)	0.836
AST, IU/L	22.50 (16.8–31.65)	21.9 (16.25–28.43)	23.4 (17.5–36.0)	0.135
Albumin, g/L	34.86 (30.85–38.87)	35.17 (31.52–38.82)	34.53 (30.19–38.87)	0.2
Total bilirubin, umol/L^a^	62.59 (56.12–69.06)	62.62 (56.13–69.11)	62.56 (56.09–69.03)	0.947
*γ*-Glutamyltransferase, IU/L	28.90 (20.20–57.00)	27.9 (18.7–44.0)	32.8 (20.5–67.40	0.107
ALP, IU/L^a^	71.50 (58.20–91.50)	68.1 (56.73–83.55)	74.6 (60.25–96.2)	0.019
Creatine kinase, IU/L	46.80 (33.65–71.70)	49.3 (33.6–71.30	44.6 (33.58–73.4)	0.919
LDH, IU/L	206.90 (174.25–273.50)	198.95 (171.88–257.70)	214.6 (178.6–288.55)	0.302
CK-MB, IU/L	9.00 (7.30–12.90)	8.70 (7.25–12.45)	9.4 (7.3–13.2)	0.35
Myoglobin, ng/ml	10.23 (4.87–20.77))	9.67 (4.97–21.98)	11.56 (4.81–20.32)	0.652
Hypersensitive troponin I, ng/ml	0.01 (0.01–0.02)	0.01 (0.01–0.18)	0.01 (0.01–0.02)	0.729
BNP, pg/ml	12.9 (0.01–62.59)	0.01 (0.01–43.35)	20.4 (0.01–66.24)	0.065
Creatinine, umol/L^a^	67.20 (56.70–79.95)	64.60 (55.10–74.50)	68.1 (57.65–88.78)	0.013
Urea nitrogen, mmol/L^a^	4.78 (3.96–5.90)	4.64 (3.82–5.43)	5.12 (4.08–6.6)	0.003
UA (uric acid), umol/L^a^	269.00 (209.00–328.00)	255.5 (209.0–293.75)	286 (208–344.5)	0.034
D-dimer, mg/L	0.69 (0.37–1.79)	0.79 (0.34–2.50)	0.69 (0.38–1.64)	0.823
PT, s	12.84 (12.2–13.70)	13.02 (12.27–13.64)	12.79 (12.12–13.93)	0.931

*Electrolyte, mmol/L*				
Na^+a^	141.60 (139.20–143.40)	141.4 (138.65–143.40)	141.7 (139.35–143.33)	0.726
Cl^−a^	105.20 (102.75–107.48)	105.5 (103.58–107.63)	104.85 (102.48–107.3)_	0.268
K^+^	4.33 (3.98–4.65)	4.4 (4.1–4.77)	4.2 (3.83–4.57)	0.002
Ca^2−^	2.09 (2.00–2.16)	2.11 (2.02–2.17)	2.07 (1.99–2.16)	0.154
*Radiographic findings*				
Ground-glass opacity	209 (74.1)	90 (63.8)	119 (84.4)	<0.001
Consolidation	18 (6.4)	3 (2.1)	15 (10.6)	0.004
Mottling opacity	205 (72.7)	78 (55.3)	127 (90.1)	<0.001
Bilateral	271 (96.1)	136 (96.5)	135 (95.7)	0.756
Unilateral	8 (2.8)	3 (2.1)	5 (3.6)	0.727
Unknown	1 (0.4)	1 (0.4)	0	
Normal	2 (0.7)	1 (0.7)	1 (0.7)	
Percentage of PIV, median (IQR), %	5.10 (1.30–12.10)	4.55 (1.15–11.78)	5.8 (1.3–12.3)	0.017

^a^Mean±SD. IQR: interquartile range; IL-6: interleukin-6; PCT: procalcitonin; ALT: alanine aminotransferase; AST: aspartate aminotransferase; PT: prothrombin time; ALP: alkaline phosphatase; LDH: lactate dehydrogenase; BNP: brain natriuretic peptide; CK-MB: creatine kinase isoenzyme; UA: uric acid; Na^+^: sodium ion; CL^−^: chloride ion; K^+^: potassium ion; Ca^2+^: calcium ion; PIV: pulmonary infection volume.

**Table 3:** Antihypertensive treatments of patients with COVID-19 and hypertension.

	All (*n* = 151)	Nonsevere patients (*n* = 106)	Severe patients (*n* = 45)	*p* value
Classification of etiology				
Primary	149 (98.7)	104 (98.1)	45 (100)	0.881
Secondary	2 (1.3)	2 (1.9)	0	

Duration of hypertension, median (IQR), years	10 (6–20)	10 (6–13)	15 (9–22.5)	0.065
Grade				
1	11 (7.3)	10 (9.4)	1 (2.2)	0.175
2	34 (22.5)	21 (19.8)	13 (28.9)	0.222
3	20 (13.2)	15 (14.2)	5 (11.1)	0.614
Unknown	88 (58.3)	60 (56.6)	28 (62.2)	0.522

Antihypertensive therapies previously				
CCBs	85 (56.3)	61 (57.5)	24 (53.3)	0.094
ACEIs/ARBs	30 (19.9)	26 (24.5)	4 (8.9)	0.031
ACEIs	9 (6.0)	6 (5.7)	3 (6.7)	0.811
ARBs	21 (13.9)	20 (18.9)	1 (2.2)	0.007
*β*-Blockers	19 (12.6)	15 (14.2)	4 (8.9)	0.373
Anticoagulant drugs	16 (10.6)	13 (12.3)	3 (6.7)	0.238
Statins	10 (6.6)	7 (6.6)	3 (6.7)	0.988
None	19 (12.6)	12 (11.3)	7 (15.6)	0.473
Diuretics	7 (4.6)	4 (3.8)	3 (6.7)	0.426
Unknown	14 (9.3)	9 (8.5)	5 (11.1)	0.76

IQR: interquartile range; CCBs: calcium channel blockers; ACEIs: angiotensin-converting enzyme inhibitors; ARBs: angiotensin II type 1 receptor blockers; anticoagulant drugs: aspirin or clopidogrel.

**Table 4:** Univariate logistic regression analysis of patients with COVID-19 and hypertension.

	Univariate OR (95% CI)	*p* value
Diabetes	2.148 (1.074–4.297)	0.031
Coronary heart disease	3.009 (1.344–6.733)	0.007
Cerebrovascular disease	4.279 (1.181–15.510)	0.027
Percentage of PIV, %	1.025 (1.004–1.046)	0.021
White blood cell count, ×109/L	1.133 (1.035–1.241)	0.007
Neutrophil count, ×109/L	1.128 (1.032–1.232)	0.008
ALP, IU/L	1.007 (0.998–1.016)	0.112
Urea nitrogen, mmol/L	1.142 (1.037–1.257)	0.007
UA (uric acid), umol/L	1.003 (1–1.005)	0.028
Creatinine, umol/L	1.016 (1.004–1.027)	0.008
K^+^	0.582 (0.383–0.885)	0.011

ALT: alanine aminotransferase; OR: odds ratio; CI: confidence interval.

**Table 5:** Multivariate logistic regression analysis of patients with COVID-19 and hypertension.

	Multivariate OR (95% CI)	*p* value
Diabetes	2.438 (0.824–7.210)	0.107
Coronary heart disease	5.281 (1.462–19.076)	0.011
Cerebrovascular disease	5.661 (0.952–33.662)	0.057
Percentage of PLV, %	0.994 (0.963–1.026)	0.71
Neutrophil count, ×10^9^/L	1.471 (1.183–1.828)	0.001
Urea nitrogen, mmol/L	0.994 (0.795–1.244)	0.961
UA (uric acid), umol/L	1.003 (0.998–1.008)	0.247
Creatinine, umol/L	1.018 (0.994–1.004)	0.149
K^+^	0.273 (0.134–0.555)	<0.001

OR, odds ratio; CI, confidence interval.

**Table 6:** Univariate and multivariate logistic regression analysis of more severe patients with COVID-19 and hypertension.

	Univariate OR (95% CI)	*p* value	Multivariate OR (95% CI)	*p* value
Percentage of PIV, %	1.093 (1.049–1.138)	<0.001	1.084 (1.035–1.135)	0.001
ARB or ACEI	0.299 (0.097–0.923)	0.036		
CK-MB, IU/L	1.141 (1.052–1.237)	0.001		
Age, years	1.061 (1.018–1.105)	0.005		
Ca^2+^, mmol/L	0.042 (0.002–0.704)	0.028		
Platelet count, ×10^9^/L	0.995 (0.991–0.999)	0.01		

Blank cells indicate no statistical significance. OR: odds ratio; CI: confidence interval; PIV: pulmonary infection volume; Ca^2+^: calcium ion; ACEI: angiotensin-converting enzyme inhibitors; ARB: angiotensin II type 1 receptor blockers.

## Data Availability

The data in the study are available from the corresponding authors on reasonable request.

## Supplementary Materials

Supplementary Materials Supplementary Table 1: classification of office blood pressure and definitions of hypertension grades according to the 2018 guidelines of the European Society of Hypertension. Supplementary Table 2: reference range of laboratory findings. Supplementary Table 3: univariate logistic regression analysis of more severe patients with COVID-19 and hypertension.
