# Supplementary material for: Risk Factors for COVID-19 in Patients with Hypertension
Source: Can J Infect Dis Med Microbiol. 2021 May 7;2021:5515941. doi: 10.1155/2021/5515941 (PMC8112195; doi:10.1155/2021/5515941)
Supplement: Supplementary Materials — Supplementary Table 1: classification of office blood pressure and definitions of hypertension grades according to the 2018 guidelines of the European Society of Hypertension. Supplementary Table 2: reference range of laboratory findings. Supplementary Table 3: univariate logistic regression analysis of more severe patients with COVID-19 and hypertension. [file 5515941.f1.pdf]

Supplementary table

Supplementary table 1. Classification of office blood pressure and definitions of hypertension grades according to the 2018 guidelines of the European Society of Hypertension.

Category	Systolic(mmHg)		Diastolic(mmHg)
Optimal	<120	and	<80
Normal	120-129	and/or	80-84
High normal	130-139	and/or	85-89
Grade 1 hypertension	140-159	and/or	90-99
Grade 2 hypertension	160-179	and/or	100-109
Grade 3 hypertension	⩾180	and/or	⩾110
Isolated systolic hypertension	⩾140	and	<90

Supplementary table 2. Reference range of laboratory findings.

	Reference range
White blood cell count, × 10^9/L	3.5–9.5
Neutrophil count, × 10^9/L	1.8–6.3
Monocyte count, × 10^9/L	0.1–0.6
Lymphocyte count, × 10^9/L	1.1–3.2
Hemoglobin, g/L	130–175
Platelet count, × 10^9/L	125–350
Eosinophil count, × 10^9/L	0.02–0.52
Basophils count, × 10^9/L	0–0.06
C-reactive protein, mg/L	0–4
IL-6, pg/mL	< 7
PCT, ng/mL	0–0.05
ALT, IU/L	9–50
AST, IU/L	9–60
Albumin, g/L	40–55
Total bilirubin, μmmol/L	0–26
γ-glutamyl transferase, IU/L	10–60
ALP, IU/L	45–125
Creatine kinase, IU/L	50–310
LDH, IU/L	120–250
CK-MB, IU/L	0–24
Myoglobin, ng/mL	0–80
Hypersensitive troponin I, ng/mL	0–0.04

BNP, pg/mL	0–100
Creatinine, μmmol/L	57–111
Urea nitrogen, mmol/L	3.6–9.5
UA, μmmol/L	202–416
D-dimer mg/L	0–0.55
PT, s	9.2–15
Na^{+}	137–147
Cl^{-}	99–110
K^{+}	3.5–5.3
Ca^{2+}	2.11–2.52

Supplementary table 3. Univariate logistic regression analysis of more severe patients with COVID-19 and hypertension

	Univariate OR (CI 95%)	p value
Diabetes	1.778(0.773-4.089)	0.176
Coronary heart disease	1.406(0.570-3.471)	0.46
Cerebrovascular disease	2.605(0.857-7.924)	0.092
Percentage of PLV,%	1.093(1.049-1.138)	<0.001
ARB or ACEI	0.299(0.097-0.923)	0.036
Age, years	1.061(1.018-1.105)	0.005
White blood cell count,×109/L	1.374(1.179-1.602)	<0.001
Neutrophil count,×109/L	1.632(1.343-1.984)	<0.001
Monocyte count,×109/L	0.634(0.223-1.803)	0.393
Lymphocyte count,×109/L	0.1(0.038-0.259)	<0.001
Haemoglobin, g/L	1.004(0.991-1.017)	0.544
Platelet count,×109/L	0.995(0.991-0.999)	0.01
Eosinophil count,×109/L	0(0-0.006)	0.001
Basophils count,109/L	0(0-7.328)	0.067
C-reactive protein, mg/L	1.019(1.010-1.027)	<0.001
PCT, ng/ml	1.391（1.071-1.806）	0.013
ALT, IU/L	1.004(0.992-1.015)	0.512
AST, IU/L	1.018(1.000-1.036)	0.047
Albumin,g/L	0.759(0.679-0.848)	<0.001
Total bilirubin, umol/L	1.051(0.985-1.120)	0.131
γ-glutamyltransferase,IU/L	1.005(0.998-1.012)	0.15
ALP, IU/L	1.009(0.995-1.024)	0.19
Creatine kinase, IU/L	1.004(1.000-1.008)	0.084

LDH,IU/L	1.016(1.010-1.022)	<0.001
CK-MB,IU/L	1.141(1.052-1.237)	0.001
Myoglobin, ng/ml	1.010(1.000-1.021)	0.05
Hypersensive troponin I, ng/ml	0.809(0.368-1.781)	0.599
BNP, pg/ml	1(0.999-1.001)	0.754
Creatinine, umol/L	0.999(0.994-1.0030	0.58
Urea nitrogen, mmol/L	1.007（0.969-1.046）	0.737
UA(uric acid), umol/L	0.998(0.994-1.001)	0.194
D-dimer mg/L	1.15（1.024-1.292）	0.018
PT , s	1.507（1.177-1.929）	0.001
Electrolyte, mmol/L		
Na	1.012（0.975-1.050）	0.529
K	1.337（0.820-2.181）	0.244
Cl	0.996（0.974-1.018）	0.691
Ca	0.042（0.002-0.704）	0.028
